# Supplementary material for: Imidacloprid decreases energy production in the hemolymph and fat body of western honeybees even though, in sublethal doses, it increased the values of six of the nine compounds in the respiratory and citric cycle
Source: PLoS One. 2025 Jul 1;20(7):e0320168. doi: 10.1371/journal.pone.0320168 (PMC12212523; doi:10.1371/journal.pone.0320168)
Supplement: S1File — (PDF) [file pone.0320168.s001.pdf]

Supporting information

Imidacloprid decreases the energy production in hemolymph and fat body of western honeybees even though, in sublethal doses, it increased the values of six of the nine compounds in the respiratory and citric cycle

Jerzy Paleolog 1,*, Jerzy Wilde 2, Marek Gancarz 3,4,5 and Aneta Strachecka 1

1. Department of Invertebrate Ecophysiology and Experimental Biology, University of Life Sciences in Lublin, Doświadczalna 50a, 20-280 Lublin, Poland
2. Department of Poultry Science and Apiculture, Faculty of Animal Bioengineering, Warmia and Mazury University in Olsztyn, Słoneczna 48, 10-957 Olsztyn, Poland
3. Faculty of Production and Power Engineering, University of Agriculture in Kraków, Balicka 116B, 30-149 Kraków, Poland
4. Institute of Agrophysics, Polish Academy of Sciences, Doświadczalna 4, 20-290 Lublin, Poland.
5. Center of Innovation and Research on Healthy and Safe Food, University of Agriculture in Kraków, Balicka 104, 30-149 Kraków, Poland

* Correspondence: jerzy.paleolog@up.lublin.pl; Tel.: +48-602725175

S1 Table. Chemical compounds examined in our bees and references to analytical procedures recommended by their manufacturers.

**A compound name** (abbreviation) [unit]	**A producer instruction how to evaluate concentration or activity of a given chemical compound.** *Activities were evaluated in the case of COX and UQCR whereas in the case of the remaining biochemical compounds the concentrations were considered.*
**Acetyl Coenzyme A** (Acetyl-CoA) [nmol/mg]	Acetyl Coenzyme A (Acetyl-CoA), Assay Kit https://www.mybiosource.com/assay-kits/acetyl-coenzyme-a-acetyl-coa/9719208
**Isocitrate dehydrogenase** (IDH-2) [ng/ml]	Isocitrate dehydrogenase (IDH-2), Assay kit https://www.assaygenie.com/human-idh2-isocitrate-dehydrogenase-2-elisa-kit/
**Alpha-Ketoglutarate** (AKG) [ng/ml]	Alpha-Ketoglutarate Assay Kit (Colorimetric) https://www.cellbiolabs.com/alpha-ketoglutarate-assay-kits
**Succinate** [μmol/l]	https://www.sigmaaldrich.com/PL/pl/specification-sheet/SIGMA/MAK184
**Fumarate** [μmol/l]	MAK060 Sigma-Aldrich Fumarate Assay Kit
**Nicotinamide adenine dinucleotide** (NADH2) [μmol/l]	NADH2-elisa-kit Products https://www.mybiosource.com/NADH2-elisa-kit
**Cytochrome c Oxidase,** (COX) [U/mg]	Cytochrome c Oxidase Assay Kit, https://www.sigmaaldrich.com/PL/pl/product/sigma/cytocox1
**Cytochrome c reductase** (UQCR) [U/mg]	Cytochrome c Reductase (NADPH) Assay Kit, https://www.sigmaaldrich.com/PL/pl/product/sigma/cy0100
**Adenosine triphosphate** (ATP) [nmol/mg]	ATP Assay Kit (Colorimetric/Fluorometric) (ab83355) https://www.abcam.com/products/assay-kits/atp-assay-kit-colorimetricfluorometric-ab83355.html

S2 Table. Standard deviations (SD) and standard deviations expressed as percentages of the appropriate means' values (the variability coefficient; W%) in the groups which were exposed and not exposed to imidacloprid.

TISSUE	GROUP	Acetyl-CoA [nmol/mg]		IDH-2 [ng/ml]		AKG [ng/ml]		succinate [μmol/l]		fumerate [μmol/l]		NADH2 [μmol/l]		COX [U/mg]		UQCR [U/mg]		ATP [nmol/mg]	
		SD	W%	SD	W%	SD	W%	SD	W%	SD	W%	SD	W%	SD	W%	SD	W%	SD	W%
HE	IM-0	10.4	7.8	1.62	14.6	0.74	9.0	0.59	7.4	0.67	15.6	1.03	7.2	0.17	11.7	0.09	10.7	0.67	10.6
	IM-5	7.3	8.7	1.28	9.2	0.60	5.7	0.63	6.0	0.28	4.7	1.12	18.5	0.26	10.8	0.16	14.7	0.89	24.1
	IM-200	7.5	10.2	1.09	21.7	0.49	20.0	0.40	13.2	0.21	17.0	0.93	23.3	0.13	19.9	0.04	12.6	0.30	20.1
FB	IM-0	8.35	5.3	1.4	8.1	0.78	6.3	0.67	7.2	0.67	10.6	1.01	5.5	0.14	9.7	0.09	6.9	0.75	9.0
	IM-5	5.08	4.6	0.82	4.0	0.20	1.4	0.59	4.6	0.13	1.9	0.82	9.6	0.40	11.1	0.13	8.9	0.43	8.9
	IM-200	4.59	4.5	0.75	9.0	0.51	10.3	0.52	10.5	0.31	12.8	0.51	7.5	0.06	6.5	0.09	14.8	0.45	11.8

Explanations: Hemolymph (HE). Fat body (FB). The group the bees were not given imidacloprid in their diet (ID-0). The group the bees were fed with the diet containing 5 ppb of imidacloprid (IM-5). The group the bees were fed with the diet containing 200 ppb of imidacloprid (IM-200). Acetyl Coenzyme A (Acetyl-CoA). Isocitrate dehydrogenase (IDH-2). Alpha-Ketoglutarate (AKG). Nicotinamide adenine dinucleotide (NADH2). Cytochrome c Oxidase (COX). Cytochrome c reductase (UQCR). Adenosine triphosphate (ATP). Activities were evaluated in the case of UQCR and COX whereas in the case of the remaining biochemical compounds the concentrations were considered.

S1 Figure. Response of the biochemical compound involved in the energy metabolism in bees exposed and not exposed to different doses of imidacloprid - part 1.

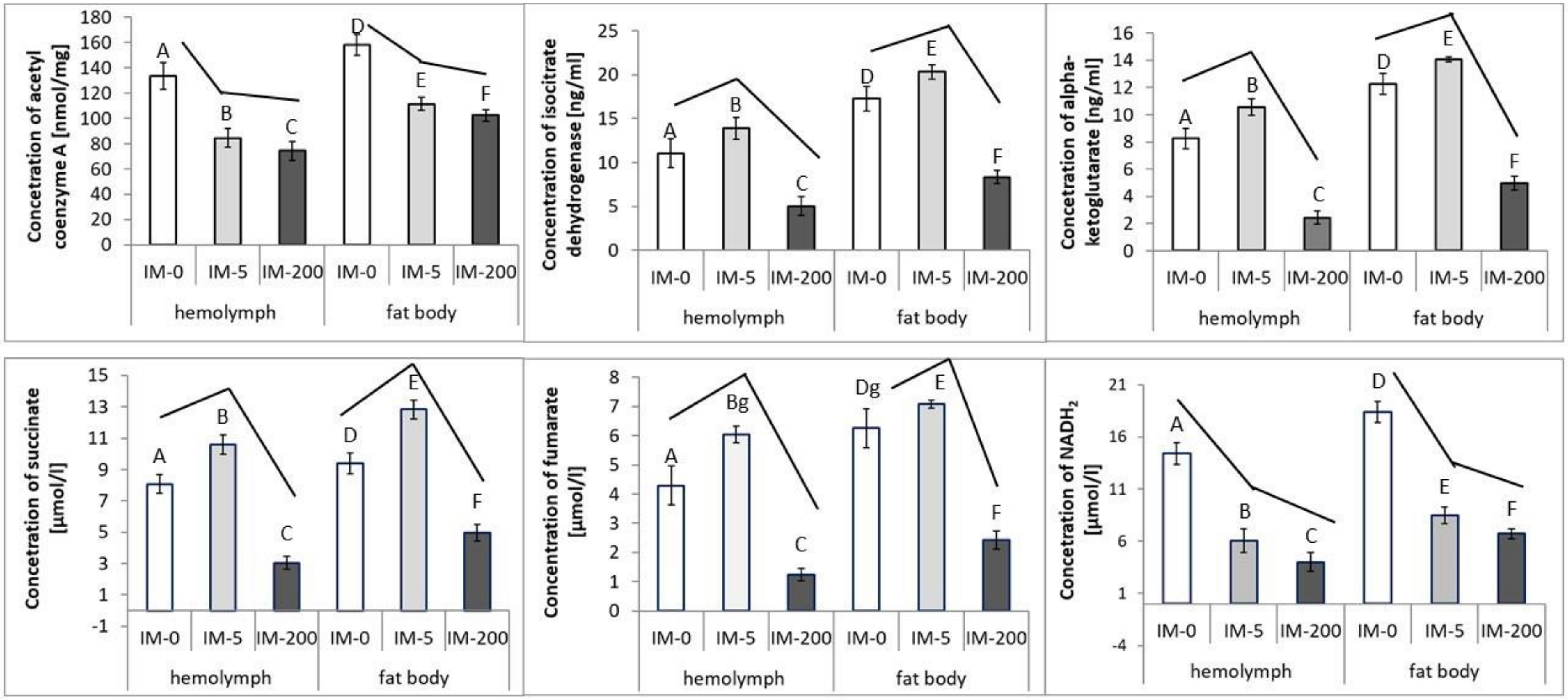

Explanation: The feeding group the bees were not given imidacloprid in their diet (ID-0). The feeding group the bees were fed with the diet containing 5 ppb of imidacloprid (IM-5). The group the bees were fed with the diet containing 200 ppb of imidacloprid (IM-200). The lines plotted above the bars nested within hemolymph and fat body tissues visualize the potential interactions tissue x feeding group within every biochemical compound separately. As they are almost the same within hemolymph and fat body the

response patter of a given compounds to the imidacloprid diets was the same in both tissues – no interactions occur. The capital letters - differences between the means are significant at $p < 0.001$. Lower case letters - differences between the means are significant at $p < 0.05$.

S2 Figure. Response of the biochemical compound involved in the energy metabolism in bees exposed and not exposed to different doses of imidacloprid- part 2.

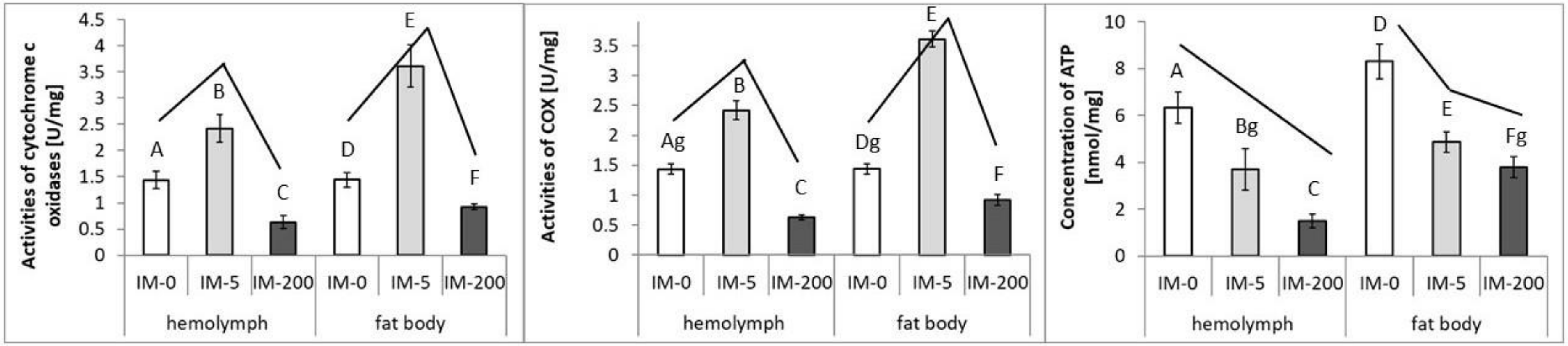

Explanation: The feeding group the bees were not given imidacloprid in their diet (ID-0). The feeding group the bees were fed with the diet containing 5 ppb of imidacloprid (IM-5). The group the bees were fed with the diet containing 200 ppb of imidacloprid (IM-200). The lines plotted above the bars nested within hemolymph and fat body tissues visualize the potential interactions tissue x feeding group within every biochemical compound separately. As they are almost the same within hemolymph and fat body the response patter of a given compounds to the imidacloprid diets was the same in both tissues – no interactions occur. The capital letters - differences between the means are significant at $p < 0.001$. Lower case letters - differences between the means are significant at $p < 0.05$.
