# Supplementary material for: Imidacloprid decreases energy production in the hemolymph and fat body of western honeybees even though, in sublethal doses, it increased the values of six of the nine compounds in the respiratory and citric cycle
Source: PLoS One. 2025 Jul 1;20(7):e0320168. doi: 10.1371/journal.pone.0320168 (PMC12212523; doi:10.1371/journal.pone.0320168)
Supplement: Attachment — Submitted filename: reviewer comments_PONE-D-25-08140_review.docx [file pone.0320168.s002.docx]

PONE-D-25-08140: Imidacloprid decreases the total energy production in western honeybees even though, in sublethal doses, it increased the values of six of the nine compounds in the respiratory and citric cycle

After reading the entire text, I realized that the authors investigated the effects of imidacloprid on the concentration or activity of compounds produced in the respiratory and citric acid cycles in the hemolymph and fat bodies of honey bee. The authors claim that they avoided directly using transcriptomics and metabolomics to reveal the molecular-level effects of neonicotinoid pesticides on bees. Instead, they used kits to measure the concentration or activity of metabolic products at the tissue level after pesticide exposure. This may be a cost-effective method that can quickly yield results. Therefore, this article has the value of being published in a scientific journal. Here, I would like to ask the author a few academic questions regarding this paper, and I hope for a discussion.

1. Why did the authors feed the colonies sugar syrup with imidacloprid rather than by rearing caged bees? I ask this question because the author does not present any data at the colony level in the article?
2. The authors mentioned in lines 145-148 that they captured nurse worker bees for laboratory analysis, but there are no analysis results concerning nurse worker bees in the results section. Why is that ?
3. Why was the exposure duration to imidacloprid set at 6 weeks instead of 5 weeks or 7 weeks?
4. What was the strength of the experimental colonies established? How many combs does each hive contain?

If the author addresses all the issues in the manuscript, then this paper is worth publishing in the PLOS ONE. I recommend the authors make major revisions.

**Title**

The original title of the authors' work is "Imidacloprid decreases the total energy production in western honeybees even though, in sublethal doses, it increased the values of six of the nine compounds in the respiratory and citric cycle." This title does not align with the actual content of the study. The authors state in the title that "Imidacloprid decreases the total energy production in western honeybees." However, the study only examined the concentration and activity of products in the metabolic processes of hemolymph and fat bodies. Metabolism in the hemolymph and fat bodies alone cannot represent the total energy of the bees. Therefore, the current title is extremely imprecise and should be revised.

**Abstract**

The elements of an abstract include background, objectives, methods, results, conclusions, and significance; please supplement the missing sections.

**Introduction**

Lines 48-49: Replace “1-(6-chloro-3-pyridylmethyl)-2nitroimino-imidazolidine” with “1-(6-chloro-3-pyridylmethyl)-2-nitroimino-imidazolidine”

Line 51: What is the full name of “EU” in “forbidden in the EU”?

Line 74: Replace “CO2” with “CO_2_”. Please check all the subscripts that appear throughout the text.

Lines 114-115: In this sentence, "While hemolymph is a tissue that has been quite often analyzed in bees, information about the metabolism of the apian fat body is fragmentary," there is no causal relationship between hemolymph and fat body. Therefore, the logical expression of this sentence is flawed.

**Materials and Methods**

Please indicate the time of the experiment, such as season and month.

**Results**

Line 222: instead of “p < 0.00001 (#) or p < 0.001 (&).” with [italic](javascript:;) “*p* < 0.00001 (#) or *p* < 0.001 (&)”.

The description of the results section is too simplistic, making it appear somewhat unclear.

**Discussion**

The discussion section needs to add subheadings to indicate which aspects the authors discussed.

**Conclusions**

Lines 421-426: This conclusion needs to be reconsidered; please revise it in conjunction with the earlier comments.

**References**

The current reference formatting style of the authors does not meet the publishing requirements of PLOS ONE. Some of the reference citations are incomplete, missing issue numbers, volume numbers, or page numbers. The formatting of journal names is inconsistent, with some using abbreviations and others using full titles. The capitalization of words in the citations is also inconsistent. The reference list needs to be reviewed and formatted consistently to meet academic publication standards. Please check the entire reference list for proper formatting.

Italicize genus names:

line 442: “Varroa destructor”

line 443: “Apis Mellifera”

line 494: “Drosophila melanogaster”

line 497: “Apis Mellifera”

line 500: “Apis Mellifera”

line 512: “Apis Mellifera”

line 520: “Apis Mellifera”

line 524: “Apis Mellifera”

line 528: “Apis Mellifera”

line 532: “Apis Mellifera”

line 544: “Drosophila”

line 576: “Apis Mellifera”

line 589: “Apis Mellifera”

line 590: “Nosema ceranae”

line 590: “Apis cerana”

Please check the entire reference list for proper formatting.

Supporting information

What do the capital letters at the top of the bar chart in Figure S1 and S2 represent? What does the line connecting the bars indicate?
