# Supplementary material for: Imidacloprid decreases energy production in the hemolymph and fat body of western honeybees even though, in sublethal doses, it increased the values of six of the nine compounds in the respiratory and citric cycle
Source: PLoS One. 2025 Jul 1;20(7):e0320168. doi: 10.1371/journal.pone.0320168 (PMC12212523; doi:10.1371/journal.pone.0320168)
Supplement: Attachment — Submitted filename: Response to Reviewers.docx [file pone.0320168.s005.docx]

Response to Reviewers and Academic Editor – general remarks

PONE-D-25-08140

Imidacloprid decreases the total energy production in western honeybees even though, in sublethal doses, it increased the values of six of the nine compounds in the respiratory and citric cycle

PLOS ONE

**1. Response to the Editor general comments:**

After careful consideration, we feel that it has merit but does not fully meet PLOS ONE’s publication criteria as it currently stands…….

Authors: The manuscript has been reedited to match to the “Submission Guidelines”. All points raised during the review process have been duly considered – the manuscript has been completed and reworded. Additional references have been added as well.

If you would like to make changes to your financial disclosure, please include your updated statement in your cover letter.

Authors: The following statement has been added to the financial disclosure: *"The funders had no role in study design, data collection and analysis, decision to publish, or preparation of the manuscript."*

If applicable, we recommend that you deposit your laboratory protocols in protocols.io to enhance the reproducibility of your results. Protocols.io assigns your protocol its own identifier (DOI) so that it can be cited independently in the future. ….. Additionally, PLOS ONE offers an option for publishing peer-reviewed Lab Protocol articles, which describe protocols hosted on protocols.io..

Authors:

a) The analytical protocols of the kits producers were employed in this study. The appropriate links to the producers’ websites are given in the S1_Table within the Supporting Information.

b) The field and sampling procedures have already been published by us and the appropriate papers are cited in the manuscript – respectively [19], [41] and [37]

Journal requirements:

When submitting your revision, we need you to address these additional requirements.

1. Please ensure that your manuscript meets PLOS ONE's style requirements,….

Authors: Manuscript has been duly reedited to meet PLOS ONE's style requirements

2. Thank you for stating the following financial disclosure: If this statement is not correct you must amend it as needed. Please include this amended Role of Funder statement in your cover letter; we will change the online submission form on your behalf.

Authors: the financial disclosure has been corrected as follows:

“AS; The founding no. is: LKE.SUBB.WLE.22.058 by Ministry of National Education of the Republic of Poland, via University of Life Science in Lublin, Poland. The funders had no role in study design, data collection and analysis, decision to publish, or preparation of the manuscript.”

The proper information is included within the Cover Letter

3. When completing the data availability statement of the submission form, you indicated that you will make your data available on acceptance. We strongly recommend all authors decide on a data sharing plan before acceptance, as the process can be lengthy and hold up publication timelines. Please note that, though access restrictions are acceptable now, your entire data will need to be made freely accessible if your manuscript is accepted for publication. This policy applies to all data except where public deposition would breach compliance with the protocol approved by your research ethics board. If you are unable to adhere to our open data policy, please kindly revise your statement to explain your reasoning and we will seek the editor's input on an exemption. Please be assured that, once you have provided your new statement, the assessment of your exemption will not hold up the peer review process.

Authors: We have confirmed that our data are available at [https://repod.icm.edu.pl/dataset.xhtml?persistentId=doi:10.18150/MMKUSE.](https://repod.icm.edu.pl/dataset.xhtml?persistentId=doi:10.18150/MMKUSE.%20)

However the following DOI number have been activated during the review process: <https://doi.org/10.18150/MMKUSE>. Therefore, the better is to use it. We have informed this within the “Cover letter – revision”.

4. Please include captions for your Supporting Information files at the end of your manuscript, and update any in-text citations to match accordingly. Please see our Supporting Information guidelines for more information: <http://journals.plos.org/plosone/s/supporting-information.>

Authors: The proper captions have been added in the form of the Level 2 headings

**2. Response to the Reviewer #2 included within the “Annotated article for the attention of the authors” (made within a .pdf file of the manuscript)**

**Abstracts:**

**Reviewer:** Line 29 and 34 – Authors: the symbols have been corrected and defined

**Introduction:**

**Reviewer:** Line 114 and 119 – Authors: wording has been corrected exactly as the reviewer suggested

**Reviewer:** Line 116 –

Authors: one reference [32] that is a review paper concerning the knowledge about the latest researches on the apian fat body has been added.

**Reviewer:** Line 119 – Authors: wording has been corrected exactly as the reviewer suggested

**Materials and methods:**

**Reviewer:** Line 128 –

Authors: the proper items of information about this when the experiment took place (year, months, periods) was added in three places of M&M section.

**Reviewer:** Line 149 – Authors: wording has been changed exactly as the reviewer suggested

**Reviewer:** Line 182 –

Authors: PCA it is not the same like ANOVA. They complement each other. The values given in ANOVA and PCA do not have to be the same. So, information given by us is proper. PCA does not take into account the experimental variables and factors of the database of a given experiment - as the ANOVA does. Instead of this, PCA expresses the variability of the database on a scale from -1 to +1 and examines which, hypothetical, factors influence such variability in the greatest extent creating a series of hypothetical variance components ranking them from the most important to the least important one (PC1, PC2, ..... PCn; principal component analysis). Usually, we consider 2 (two-dimensional graph) or 3 (three-dimensional graph) of them being of the most importance; In this experiment we have considered two principal components (PC1 and PC2). Using this background, PCA analyses impact of the real experimental variables/factors. The further they are plotted from the value 0, the greater their influence is (share in the principal components). The closer they are plotted each other, the higher are the correlations between them, and so on. ……….The appropriate explanation has been added in the text and the text was a little bit reedited for better understanding.

**Results and discussion:**

**Reviewer:** Line 188 –

Authors: This not occurred in the control groups as the Reviewer suggested. It is misunderstanding. To clarify this we have changed the wording in the lines 188-190. The current version is: “*The patterns of response to imidacloprid was the same in hemolymph and the fat body irrespective of the diet type. On the other hand, the response patterns were different and even opposite in IM-200 and IM-5 irrespective of the tissue type ……”*. It should be clear for a reader now.

**Reviewer:** Line 194-195 –

Authors: Supporting Information - S1 Fig and S2 Fig.

Meaning of the letters above the bars were explained within the Figs’ legends. In S1 Fig, "Concetration" has been replaced by "Concentration" (vertical axes). Compound nomenclature has been adjusted to match that applied in the revised version of the manuscript. The meaning of the lines above the bars (S1 Fig and S2 Fig) has been explained. The expression “(compare the interaction plots at S1 Fig and S2”) has been reworded to (“compare the interaction lines above the bars nested within a given compound and tissue at S1 and S2 Figs)”. Some part of the manuscript has been moved up. The proper explanation has been added.

These changes stay also in line with remarks of Reviewer#2 as well.

**Reviewer:** Line 202-204:

Authors: The sentence has been reworded as follows: “Furthermore, all the chemical compounds are plotted very near the circle that defines the area of maximum influence (it crossing │1│of PC1 and PC2), so all of them were very strongly affected by the diet types.”

**Reviewer:** Line 206-210 –

Authors: Nomenclature and description of this quadrant of this graph has been corrected. The manuscript has been corrected.

**Reviewer:** Line 234-328 –

Authors: This part of manuscript has been reworded according the Reviewer suggestions. Nomenclature of the compounds was corrected (also within the Tables).

**Reviewer:** Line 249: Authors: Nomenclature was corrected

**Reviewer:** Line 256, 262: Authors: All figures have been corrected where necessary.

**Reviewer:** Line 281:

Authors: This has been reworded as follows: the component values were plotted very near the circle of the value │1.0│ of PC1 and PC2

**Reviewer:** Line 282: Authors: This has been corrected according the Reviewer suggestion

**Reviewer:** Line 289:

Authors: the following has been added; “Therefore, it can be assumed that our bees were facing imidacloprid during entire experiment period, not only by the applied diets but also but the comb storages.”

**Reviewer:** Line 300: Authors: This has been corrected according the Reviewer suggestion

**Reviewer:** Line 311:

Authors: The sentence after correction is as follows: “that had positive PC2 and negative PC1 values.”

**Reviewer:** Line 316: Authors: The typing error has been corrected

**Reviewer:** Line 223:

Authors: The sentence has been reworded according the Reviewer suggestion as follows: “three of them which are those of the greatest importance....”

**Reviewer:** Line 340: Authors: The typing error has been corrected

**Reviewer:** Line 366: Authors: the word “metabolic” has been added as Reviewer has suggested

**Reviewer:** Line 374: Authors: The typing error has been corrected

**Reviewer:** Lines 392 to 401: Authors: this part was reedited and reworded

**Reviewer:** Lines 408- 409

Authors: The sentence after rewording is: “It is the next bad information news for beekeepers, who keep bees dwelling in an environment where nicotinoids occur that….

**Reviewer:** Lines 412: Authors: The typing error has been corrected.

**Reviewer:** Line 419: Authors: The expression after rewording is: "possibly also influencing"

**Reviewer:** Line 422 Authors: word “influences” has been added

**Reviewer: - References ……**

Authors: references were completely reedited

**2. Response to the Reviewer #1 (made within the WORD review file)**

**Reviewer: why did the authors feed the colonies sugar syrup with imidacloprid rather than by rearing caged bees? I ask this question because the author does not present any data at the colony level in the article?**

Authors:

1.The information about the colonies’ strength and a syrup consumption are given in the manuscript.

2. The following paragraph has been added to the discussion:

“Laboratory cage tests are applied in honeybee research to make it possible to control environment factors. However, the intra colony environment may affect many traits, particularly functional ones. Consequently, results obtained in field experiments may differ from those obtained in cages, even if individual, biochemical traits of a single worker are considered [38]. For instance, he cage environment had a destabilizing effect on the natural protease inhibitor system and decreased the worker resistance to microorganisms, which was not the case with the hive environment [39]. Hence, results of cage-based analyses of non-specific apian resistance should be treated with caution when used in reference to hive bees. Cresswell [40] also pointed out the problems about the relevance of laboratory-based results in toxicological research, and consequently considered “possibilities of providing a bridge between laboratory bioassay testing and full field experimentation”. Therefore, in this research, we decided to study respiratory and citric cycle compounds in workerbees exposed to imidacloprid in field assays, as we already had done in our previous studies [19, 41], assuming that honeybees dwell in colonies kept in the natural conditions rather and not in laboratory cages. We hoped that the results obtained in this way would be more applicable for the practical apiculture.”

The added references:

[38] Dziechciarz P., Borsuk G., Olszewski K. (2019). Prospects and validity of laboratory cage tests conducted in honeybee research part one: main directions of use of laboratory cage tests in honeybee research. J. Apic. Sci. 63, (2), 201-207.]

[39] Strachecka A. Paleolog J., Borsuk G., Olszewski K. Grzywnowicz K, Gryzińska M. (2011) Body-surface protease inhibitors in cage and hive Apis mellifera L. Acta Sci. Pol., Zootechnica 10 (4), 125–132

[40] Cresswell, J.E. A meta-analysis of experiments testing the effects of a neonicotinoid insecticide (imidacloprid) on honey bees. Ecotoxicology 20, 149–157 (2011). https://doi.org/10.1007/s10646-010-0566-0

**Reviewer: The authors mentioned in lines 145-148 that they captured nurse worker bees for laboratory analysis, but there are no analysis results concerning nurse worker bees sampled from the experimental colonies were presented in this section. Why is that ?**

Authors: It is some kind of misunderstanding. The only results concerning nurse workerbees have been shown in the “Results” section. To solve the problem we changed the following:

a). The aim (Introduction) has been changed and its current wording is: “The aim of this study was to compare the concentrations/activities of the above compounds in workerbees captured from the colonies fed with a diet containing 200 ppb, 5 ppb or 0 ppb of imidacloprid. Both the workerbee hemolymph and fat bodies were assayed.”

b). Paragraph 2.3. of M&M has been changes and is wording is as follows: “The concentrations of acetyl-CoA, IDH-2, AKG, succinate, fumarate, NADH2 and ATP, as well as the activities of COX and UQCR were evaluated in the hemolymph and fat-body supernatant of workerbees sampled from the experimental colonies following the producer instructions for each specific kit (S1 Table).”

c) Finally, the first part of the results section has been reedited and its wording is: “The values of compounds involved in energy-metabolism were higher in the fat body than in the hemolymph of workerbees sampled from our experimental colonies, whereas the compound variations were markedly higher in the hemolymph. This was particularly seen in the IM-0 group, in which the workers were not exposed to imidacloprid (Table 1).

d) Consequently the table and figures titles have been changed in agreement with the pints a, b, and c.

e) The expression “laboratory analysis” has bee replaced by the expression “biochemical analysis”, which seems to be more adequate. The expression “laboratory analysis” may suggest the cage test

**Reviewer: Why was the exposure duration to imidacloprid set at 6 weeks instead of 5 weeks or 7 weeks?**

Authors: The following explanation has been added in M&M (the line 148): “The 6-week feeding period guaranteed that each sampled nurse workerbee received imidacloprid throughout its whole life from the moment the egg, she emerged, had been laid. On the other hand foragers are exposed to numerous external factors, hence analyzing of the nurse worker bees was justified.”

**Reviewer: What was the strength of the experimental colonies established? How many combs does each hive contain?**

Authors: The text beginning from the line 126 has been reedited as follows: “The synthetic colonies of similar strength, as well as worker and brood structure, were set up in northeastern Poland (19.53 E, 53.50 N) in June 2024. Each of these colonies fully populated a one-box hive with ten 360 mm x 260 mm frames and was headed by an egg laying one-year-old purebred queen. All the queens belonged to the same Apis mellifera carnica commercial stock and were all obtained from the same mother-queen. In the first ten days of June, after removing the hive food-stores, the colonies were given sugar/water syrup (5:3 w/w)”

**Title**

**Reviewer:** The original title of the authors' work is "Imidacloprid decreases the total energy production in western honeybees even though, in sublethal doses, it increased the values of six of the nine compounds in the respiratory and citric cycle." This title does not align with the actual content of the study. The authors state in the title that "Imidacloprid decreases the total energy production in western honeybees." However, the study only examined the concentration and activity of products in the metabolic processes of hemolymph and fat bodies. Metabolism in the hemolymph and fat bodies alone cannot represent the total energy of the bees. Therefore, the current title is extremely imprecise and should be revised.

Authors: The new Title is: “Imidacloprid decreases the energy production in hemolymph and fat body of western honeybees even though, in sublethal doses, it increased the values of six of the nine compounds in the respiratory and citric cycle”

**Reviewer:** The elements of an abstract include background, objectives, methods, results, conclusions, and significance; please supplement the missing sections.

Authors: The entire abstract has been reedited. Please see the new version of the manuscript.

**Introduction**

**Reviewer:** Lines 48-49: Replace “1-(6-chloro-3-pyridylmethyl)-2nitroimino-imidazolidine” with “1-(6-chloro-3-pyridylmethyl)-2-nitroimino-imidazolidine”

Authors: It has been replaced

**Reviewer:** Line 51: What is the full name of “EU” in “forbidden in the EU”?

Authors: EU has been replaced by European Union.

**Reviewer:** Line 74: Replace “CO2” with “CO2”. Please check all the subscripts that appear throughout the text.

Authors: Corrected; CO2 to CO_2_. All the subscripts have been checked

**Reviewer:** Lines 114-115: In this sentence, "While hemolymph is a tissue that has been quite often analyzed in bees, information about the metabolism of the apian fat body is fragmentary," there is no causal relationship between hemolymph and fat body. Therefore, the logical expression of this sentence is flawed.

Authors: The sentence was divided into parts and reworded as follows:

“Finally hemolymph is a tissue that has been quite often analyzed in bees, since as “a carrier” it contact and supply many apian tissues and participate in their metabolism. Therefore it can be treated as a good reference for other tissues. On the other hand, information about the metabolism and functions of the apian fat body is fragmentary, however views on its role has been substantially changing during the last decade [32]. Consequently, we wanted to fill in this gap in the knowledge about the effects of imidacloprid on the most important processes of cellular respiration, energy metabolism pathways and total energy in the honeybee fat body in comparison with hemolymph.”

**Materials and Methods**

**Reviewer:** Please indicate the time of the experiment, such as season and month.

Authors: This has been indicated in two places of M&M

**Results**

**Reviewer:** Line 222: instead of “p < 0.00001 (#) or p < 0.001 (&).” with italic “p < 0.00001 (#) or p < 0.001 (&)”.

Authors: This has been changed into italic

**Reviewer:** The description of the results section is too simplistic, making it appear somewhat unclear.

Authors: The Results section has been reedited, the additional information has been also added within the manuscript as well as within the Supporting Information. These changes particularly concerns the PCA analysis

**Discussion**

**Reviewer:** The discussion section needs to add subheadings to indicate which aspects the authors discussed.

Authors: The subheadings has been added

**Conclusions**

**Reviewer:** Lines 421-426: This conclusion needs to be reconsidered; please revise it in conjunction with the earlier comments.

Authors: The wording of the conclusion has been changed and is as follows:

“1.	Imidacloprid may impair of the fat body energy metabolism and energy supplies. In this way we have revealed one more mechanism, by which neonicotinoids may deteriorate functioning of this tissue, and consequently, the body-maintenance of honeybees when facing harmful, anthropogenic environments. This point of view can be confirmed by the same changes observed in hemolymph.”

**References**

**Reviewer:** The current reference formatting style of the authors does not meet the publishing requirements of PLOS ONE. Some of the reference citations are incomplete, missing issue numbers, volume numbers, or page numbers. The formatting of journal names is inconsistent, with some using abbreviations and others using full titles. The capitalization of words in the citations is also inconsistent. The reference list needs to be reviewed and formatted consistently to meet academic publication standards. Please check the entire reference list for proper formatting.

Italicize genus names

Please check the entire reference list for proper formatting.

Authors: The entire reference list has been reedited and corrected including the genus names over the whole manuscript

**Supporting information**

**Reviewer:** What do the capital letters at the top of the bar chart in Figure S1 and S2 represent? What does the line connecting the bars indicate?

Authors: The missing explanations and additional information were added
