# Supplementary material for: Imidacloprid decreases energy production in the hemolymph and fat body of western honeybees even though, in sublethal doses, it increased the values of six of the nine compounds in the respiratory and citric cycle
Source: PLoS One. 2025 Jul 1;20(7):e0320168. doi: 10.1371/journal.pone.0320168 (PMC12212523; doi:10.1371/journal.pone.0320168)
Supplement: Attachment — Submitted filename: Response_to_Reviewers_auresp_2.docx [file pone.0320168.s006.docx]

PONE-D-25-08140R1

Imidacloprid decreases the energy production in hemolymph and fat body of western honeybees even though, in sublethal doses, it increased the values of six of the nine compounds in the respiratory and citric cycle

PLOS ONE
**Authors:**

A small correction of the title was made by the Editor as follows:

“Imidacloprid decreases the energy production in the hemolymph and fat body of western honeybees even though, in sublethal doses, it increased the values of six of the nine compounds in the respiratory and citric cycle

**Journal Requirements:**
Please review your reference list to ensure that it is complete and correct. If you have cited papers that have been retracted, please include the rationale for doing so in the manuscript text, or remove these references and replace them with relevant current references. Any changes to the reference list should be mentioned in the rebuttal letter that accompanies your revised manuscript. If you need to cite a retracted article, indicate the article’s retracted status in the References list and also include a citation and full reference for the retraction notice.

**Authors:** The reference list (chapter “References”) in the first submission of our manuscript contained 53 references. The reference list submitted in our revised manuscript (MAJOR REVISION) contained 56 references because the following 3 references were added in response to the doubts of Reviewer #2:

- [38] Dziechciarz P., Borsuk G., Olszewski K. (2019). Prospects and validity of laboratory cage tests conducted in honeybee research part one: main directions of use of laboratory cage tests in honeybee research. J. Apic. Sci. 63, (2), 201-207.]
- [39] Strachecka A. Paleolog J., Borsuk G., Olszewski K. Grzywnowicz K, Gryzińska M. (2011) Body-surface protease inhibitors in cage and hive Apis mellifera L. Acta Sci. Pol., Zootechnica 10 (4), 125–132
- [40] Cresswell, J.E. A meta-analysis of experiments testing the effects of a neonicotinoid insecticide (imidacloprid) on honey bees. Ecotoxicology 20, 149–157 (2011). <https://doi.org/10.1007/s10646-010-0566-0>

The Reviewer #2 has accepted this changes.

No references were replaced or retracted, although the order of citing publications was changed a little due to corrections made in response to the Reviewers' #1 and #2 comments.

During the second MINOR REVISION no references were added, replaced or retracted, as well as the order of citing did not changed.

**Reviewer #3: General Comments:**

**Rev. #3:** The premise of the study seems solid, and the methods and analysis seem fine, from my knowledge of this area. The authors have correctly identified the importance of this study - adding to existing information on an important topic (imidacloprid effects on bees), while also providing a valuable example on the more general principle of hormesis.
Further, the suggestions of the original reviewers appear to have been addressed - either the manuscript was changed as requested (usually), or the authors responded with justification for their approach. On that basis, the manuscript meets the requirements outlined by the original reviewers.

**Rev. #3:** I believe the main area of weakness is in some of the communication - the writing is awkward in many places. Some corrections were pointed out by the original reviewers and corrected. However, there is still significant text that would benefit greatly from rewording.

**For instance, iL101-3** “Hence, more studies are necessary to answer whether the energy supply decreased by the sublethal doses of the pesticide or just hormesis may occur in this case?” is grammatically difficult to understand.

**Authors:** The sentence has been reworded as follows:

*“Consequently, more studies are necessary to answer whether body energy supply is decreased by sublethal doses of the pesticide or whether such doses can also increase the supply as a result of hormesis.”*

**Rev. #3:** There are many instances like this. It was not possible as a reviewer to suggest corrections for all of these. I suggest engaging a writing editor who can dedicate some time to making those corrections.

**Authors:** The licensed editor has made corrections that are within a marked-up copy of our manuscript labeled 'Revised Manuscript with Track Changes'. The corrections of the editor are marked by “HP” (blue).

**Reviewer #3: Specific comments**

**Rev. #3:** L30-31. Example of awkward wording here. Stating “The goal of the study…” rather than just bringing up a question, would help in the comprehension of the study.

**Authors:** The sentence has been reworded as follows:

**“***Therefore, our goal was to answer which of these two phenomena occurs in the hemolymph/fat body and at what doses of imidacloprid*”.

**Rev. #3:** L70-76. This can be shortened, the first two sentences for instance are very general statements that we should assume the reader knows already.

**Authors:** These sentences have been deleted.

**Rev. #3:** L84. The paragraph starting here is very long, and in fact is addressing two separate questions or points. The first has to do with conflicting results or responses when low doses of IM are used, and the second is how the response is measured (transcriptosome, in most cases). Split up these paragraphs. In the first paragraph, be clear the purpose in mentioning these various studies - that there are variable results that point towards a complicated response to IM.

**Authors:** This paragraph has been splinted into two different once. In the first one the following construction has been applied:

*“There is, however, a problem with the exposition of honeybees to sublethal, residual, field-relevant doses of imidacloprid (about 5 ppb), as different results of the exposition were obtained in different studies. This implies a yet incompletely explored complex response to this pesticide. On the one hand, unlike the higher doses, which have consistently proven to be highly detrimental, they ………..”*

**Rev. #3:** L150 There are two terms used here that are slightly different - sublethal and adverse - not mutually exclusive. Is the 5ppb adverse in some way, even though sublethal? And is the 200 ppb concentration also sublethal? It would be clearer to refine that terminology.

**Authors:** This part of manuscript has been reworded and developed as follows:

*“This concentration sometimes turn out to be adverse for honeybee colonies either impairing the physiology of an individual bee or weakening entire colonies – particularly in a long-term perspective [15, 16, 28]. Notably, its negative symptoms may be hidden. On the other hand, the concentration of 200 ppb is considered harmful or severely harmful and even lethal sometimes, both at level of a single bee or the colony. Its negative symptoms are usually violent and visible in the short-term perspective [23,33–35].”*

**Rev. #3:** L227. This is, I Assume, subtext for the table, and the paragraph after this then continues the main text of the results.

**Authors:** Yes, it is subtext for the table, in fact the legend (Explanations) for tis table.

Rationale: The “Results” chapter is short and consists of two parts. The first concentrate on general analysis, and the second one, on PCA results. Most of information is included within the Table and Figs (with their explanations), but not in the contents of the "Results" manuscript. Consequently, we have placed the Table after the first part, where it was cited in, but all Figs were placed after the second, last part, which in fact, constituting only a short paragraph. Please note that legends and captions for the Table and Figures take up almost as much space as the “Results” manuscript contents. Therefore, such an arrangement to allow a reader to read this short text without long brakes – “having it in almost “one piece”. Reviewer 1 and 2 haven’t addressed no comments to such an arrangement, so it seems that they accepted it. Of course the PLOS technical editor will make the final decision.

**Rev. #3:** Fig 1,2 . In both of these, it seems like showing some measure of variability and statistical significance on the graph is needed. As I interpret it currently, it only shows the mean difference (as a ratio), and does not indicate on the graph which are statistically significant.

**Authors:** We suggest not to show either measures of variability or the letters pointing the statistical significance within Fig 1,2. Instead of this we have completed the information given in “Explanations” for these Figs, as we agree with the Rev 3 that a reader should obtain some statistical information here. Now, this part of the “Explanations” stands as follows:

*Each of these differences was significant at p < 0.001 – if they were not, they could not be considered here. The differences between IM-5 and IM-200 were also significant at p < 0.05 when compared within each of the compound separately. Detailed information about the statistical characteristics, including variability levels, is available in S1, S2 Figs and S2 Table, as well as in Table 1.”*

Rationale: The values plotted at Figs 1, 2 aren’t original data obtained in our experiment, but are result of their transformation. We subtracted the control group mean from the mean of an adequate experimental group obtaining its deviations from the control. Consequently, we have used averages, but not the source data during the further calculations. This procedure was performed for each chemical compound separately, and therefore the results cannot be compared or plotted at one figure, as different compounds have completely different scales and measurement units. Therefore, next transformation was performed (data standardization) to express each of the above differences as its percentage for the mean of the adequate control group. In this way we obtained the transformed data that have no units and are expressed in the same scale (percentage). Now one was able to compare practically everything within one figure. However, as a result of such a transformation variability is changed, so plotting it, or performing statistical comparisons, do not make sense. Although a different procedure can be used enabling the statistical comparisons, but then, the next transformation (arcsin x) have to be performed. One way to another, we will have to analyze the double transformed data and the obtained information may be confusing.

Secondly, variability of the control groups is shaped by the external factors in different ways in the case of different chemical compounds. The procedure described above allow to better show (“extract”) the effect of the experimental factor (equalizing control to 0), as well as comparing all chemical compounds at the same plot. This help to avoid the information noise connected with analyzing of many plots (separate plot for every compound) focusing on the central message of the paper. However, a reader can find all necessary statical comparisons, information about variability, and information about interactions obtained during analyzing of the original, untransformed data at S1 Table and S1, S2 Figs included within the “Supporting Information”. Table 1 preceding Figs 1 and 2 within the main manuscript also constitute an auxiliary material in this context.

**Rev. #3:** L297. This would seem to be better placed in the introduction of the article. At the least, it would read better to make the main justification (which is valid) for using field experiments earlier, and then more shortly reiterate it in the discussion.

**Authors:** This paragraph, i.e. sub-chapter entitled “The rationale for the field experiment” (current version reworded by editor), was added as the first subchapter of “Discussion” according the following suggestion of Reviewer #1:

“*why did the authors feed the colonies sugar syrup with imidacloprid rather than by rearing caged bees? I ask this question because the author does not present any data at the colony level in the article?”.*

We have added this sub-chapter (paragraph) just right here, as it isn’t involved to the rationale of the study goal, or working hypotheses, or the asked questions. Although it contains important supplementary information, is not needed for the main flow of narration/reasoning within the "Introduction." Therefore, we would like to leave this information within the “Discussion”, as we want a reader to focus on the main information flow leading her/him to the rationale of study goal when reading the "Introduction”. However, leaving the main considerations within the “Discussion“ but taking into account the Rew. #3 comments, we have decided to add a short paragraph at the end of the “Introduction”. This paragraph is as follows:

*“Many toxicological experiments on pesticides in honeybees were performed in cage or semi-cage experiments [14, 22] that allowed to better monitor/control the external conditions. However, results obtained in fully functional colonies in the field could be different. Therefore, we decided to perform a field experiment in this study, controlling environmental factors to the maximum extent.”*

**Rev. #3:** L315. This paragraph could be shifted later. If it is necessary to defend the accuracy of the data in this way, that reads more smoothly if mentioned later, after the main result of the study is discussed (which starts in the next paragraph, L342)

**Authors:** We had been urged to discussing of accuracy of original field data by the reviewers of our previous papers, since the accuracy of such a data can be unsatisfactory. Therefore, we created the second subchapter of the “Discussion” entitled “Accuracy of our data” placed after the first one entitled “The rationale for the field experiment”, since it also contained important supplementary information.

Summing up, we would like to leave these two chapters containing supplementary, important information at the beginning of the “Discussion”, if possible.

Rationale: We believe that it is better familiarize the reader with the supplementary information at the beginning of the “Discission” and only then continue the main narration. In this case a reader contionuoing the Ddiscussion move consistently from issue to issue until finally meet conclusions and the central message of our work. This seems us to be a better way of the narration flow than that, when after discussing the main issues of the paper one make the break to familiarize a reader with the supplementary issues, and subsequently, come back to the main narration flow again, to drove the conclusions.

**Rev. #3:** L448. Leave “Adaptive” off the list here. It’s not clear that hormesis is definitely found to be adaptive in all instances where it is studied. It could be a nonadaptive byproduct of other processes.

**Authors:** This has been corrected

**Rev. #3:** L477. It seems like the “biphasic” response is a major and obvious finding of the study, but is not mentioned in the conclusions.

**Authors:** The conclusion 2 has been substantially developed to reflect this issue. Now it stands as follows:

*“2. We showed that hormesis took place in most of the compounds of the respiratory and citric cycle when our bees were exposed to residual, sublethal doses of imidacloprid. The most important fact was, however, that despite this hormesis the sublethal doses of the neonicotinoid always decreased ATP production. Consequently, by interfering with energy supplies, they can harm both the honeybee health and body maintenance.”*

[While revising your submission, please upload your figure files to the Preflight Analysis and Conversion Engine (PACE) digital diagnostic tool, <https://pacev2.apexcovantage.com/>. PACE helps ensure that figures meet PLOS requirements. To use PACE, you must first register as a user. Registration is free. Then, login and navigate to the UPLOAD tab, where you will find detailed instructions on how to use the tool. If you encounter any issues or have any questions when using PACE, please email PLOS at [figures@plos.org](mailto:figures@plos.org). Please note that Supporting Information files do not need this step.

*In compliance with data protection regulations, you may request that we remove your personal registration details at any time.* [*(Remove my information/details)*](https://www.editorialmanager.com/pone/login.asp?a=r)*. Please contact the publication office if you have any questions.*
